# Piperlongumine inhibits the growth of non-small cell lung cancer cells via the miR-34b-3p/TGFBR1 pathway

**DOI:** 10.1186/s12906-020-03123-y

**Published:** 2021-01-07

**Authors:** Xinhua Lu, Chenyang Xu, Zhexuan Xu, Chunya Lu, Rui Yang, Furui Zhang, Guojun Zhang

**Affiliations:** 1 Department of Respiratory and Critical Care Medicine, The First Affiliated Hospital of Zhengzhou University, No. 1 Jianshe East Road, Zhengzhou City, 450052 Henan Province China; 2 Luoyang Orthopedic-Traumatological Hospital of Henan Province (Henan Provincial Orthopedic Hospital), Zhengzhou, 450015 China

**Keywords:** Piperlongumine, miR-34b-3p, Non-small-cell lung cancer, TGFBR1

## Abstract

**Background:**

Non-small cell lung cancer is a common type of lung cancer. Piperlongumine (PL), which is extracted from the roots of piperaceae plant, long pepper, and peppercorn, is an alkaloid amide that inhibits tumor growth and metastasis. However, whether it affects lung cancer cells remains unclear.

**Methods:**

We assessed the effects of PL on the proliferation and apoptosis of A549 and H1299 NSCLC cell lines.

**Results:**

PL was mildly toxic to normal human bronchial epithelial cells and significantly suppressed growth and facilitated apoptosis of A549 and H1299 cells. It also upregulated microRNA (miR)-34b-3p and downregulated the transforming growth factor beta type I receptor (TGFBR1). The dual-luciferase reporter assay showed that TGFBR1 is a target gene of miR-34b-3p. Silencing of miR-34b-3p or overexpression of TGFBR1 partially attenuated the effects of PL on A549 and H1299 cells.

**Conclusions:**

PL inhibits proliferation and induces apoptosis of A549 and H1299 cells by upregulating miR-34b-3p and modulating TGFBR1 signaling pathway.

**Supplementary Information:**

The online version contains supplementary material available at 10.1186/s12906-020-03123-y.

## Background

Lung cancer (LC) is a major contributing factor to the cancer-related death worldwide and has a high incidence and mortality rate [1]. The two main types of LC are small cell lung cancer and non-small cell lung cancer (NSCLC). Despite continuous improvements of medical treatment and technology, the vast majority of NSCLC patients are diagnosed with late-stage disease and the 5-year survival rate is low [2]. At present, LC treatment has rapidly developed, but there are many side effects and the prognosis still remains poor [3]. Therefore, there is an urgent need to identify new targets and methods for early diagnosis in order to improve the 5-year survival rate of patients.

MicroRNAs (miRNAs) are non-coding RNAs that regulate the expression of several genes [4, 5] through complementary binding to the 3′ untranslated region (UTR) of target gene mRNAs [6, 7]. It is reported that miRNAs participate in cancer progression via regulation of cell cycle, cell proliferation, and cell invasion etc. [8, 9]. For example, miR-221 and miR-222 inhibits the growth of LC [10, 11], and miR-493 promotes the sensitivity of cancer cells to cisplatin [12]. The abnormal expression of miRNAs in LC (high or low) may lead to tumor heterogeneity and tumor-initiating cell behaviors [13].

Piperlongumine (PL) exists naturally in nature and plays an anticancer role in a variety of tumors [14]. It has been shown to inhibit cell growth and induce apoptosis of cancer cells, such as breast cancer [15], human melanoma [16], head and neck cancer [17], human prostate cancer [18], pancreatic cancer [19], gastric cancer [20], and NSCLC [21]. Furthermore, Lad [22, 23] and Mizuno et al [24] found that PL is involved in the occurrence and development of multiple cancers through regulating different signaling pathways. Mizuno et al [24] reported that miR-34b-3p can inhibit the occurrence and development of LC.

In this study, we performed a miRNA microarray assay in NSCLC cells and found the differential expression of miR-34b-3p after PL treatment. In addition, we also assessed the effects of PL on the proliferation and apoptosis of A549 and H1299 NSCLC cell lines.

## Methods

### Cells and reagents

The NSCLC cell lines A549, H1299, H520, and SPC-A-1 were purchased from the Type Culture Collection of the Chinese Academy of Sciences (Shanghai, China). A549 and H1299 cells were transfected with lentivirus containing miR-34b-3p or NC fragment to obtain stable expression of miR-34b-3p/NC (Hanbio, Shanghai, China). The PL was purchased from Sigma (St. Louis, MO, USA), dissolved in dimethyl sulfoxide (DMSO) (Sigma) and stored at 4 °C for subsequent experiments.

### RNA extraction and qPCR

Total RNA was extracted from cells using TRIzol Reagent (Invitrogen, Carlsbad, CA, USA) according to the manufacturer’s instructions. Complementary DNA (cDNA) was generated using the High Capacity cDNA Reverse Transcription Kit (Thermo Fisher Scientific, Waltham, MA, USA). miR-34b-3p (primer sequences: F- 5′-CGGCGAATCACTAACTCCACT-3′ and R-5′-GTGCAGGGTCCGAGGT-3′) and TGFBR1 (primer sequences: F-5′-GAACTGTTTTGATTGGCATC-3′ and R-5′-AAGAAGGGACCTACACTATTT-3′) mRNA expression was determined by qPCR. U6 small nuclear RNA (primer sequences: F-5′- CGCTTCGGCAGCACATATAC-3′ and R-5′- TTCACGAATTTGCGTGTCAT-3′) and GAPDH (primer sequences: F-5′-TGCCCAGAACATCATCCCT-3′ and R-5′-TGAAGTCGCAGGAGACAACC-3′) were used as the internal reference for miR-34b-3p and TGFBR1, respectively.

### miRNA microarray

A549 cells were divided into two groups: PL treatment (10 μM PL) group and control group (0.1% DMSO). Then the miRNA microarray assay was conducted by the Shanghai Bohao Biotechnology Co., Ltd. (Shanghai, China).

### Cell transfection

Transient transfection was conducted using Lipofectamine 2000 reagent (Invitrogen) following the manufacturer’s instructions. Cells were collected for subsequent experiments at different time points after transfection.

### CCK-8 assay

A549 and H1299 cells were seeded into 96-well plates. After treatment for 0, 24, 48, 72 h, 10 μL CCK-8 reagent (Dojindo, Kumamoto, Japan) was added to each well and incubated for another 1–2 h followed by measuring the optical density values to assess cell growth.

### Cell apoptosis assay

Apoptotic cell numbers were detected by Annexin V-FITC/PI Apoptosis Detection Kit (Solarbio, Beijing, China) in accordance with the kit instructions. Annexin V-FITC and PI were added to the binding buffer and mixed well followed by being added into cells. After 30 min incubation at room temperature under dark, a flow cytometer (BD Biosciences, San Diego, CA, USA) was used to measure cell apoptosis.

### Caspase-3/7 activity assay

Caspase-3/7 activity was measured using the Apo-ONE Homogeneous Caspase-3/7 Assay Kit (Promega, Madison, WI, USA). The fluorescence (relative fluorescence units) of each well was measured using a spectrofluorometer (Thermo Fisher Scientific).

### Transwell assay

A549 and H1299 cells were cultured in 6-well plates. After treatment with PL or transfection for 24 h, 5 × 10^4^ cells were seeded in the top compartment of 8 μm pores transwell culture inserts (Corning Life Sciences, Corning, NY, USA) in the presence of serum-free medium, and the experiment was performed following the instructions. Three independent experiments were performed.

### Xenograft mouse model

Female BALB/c nude mice of 4–6 weeks old (*n* = 12) were purchased from the Beijing Vital River Laboratory Animal Technology Center (Beijing, China). Three groups of A549 cells (miR-34b-3p, NC, and non-infected) were subcutaneously injected into the dorsal scapular region of the mice at a density of 2 × 10^6^. After tumors were visible (about 1 week), the tumor-bearing nude mice were intraperitoneally injected with PL at a dose of 5 mg/kg (dissolved in 0.01% DMSO) twice per week for 3 weeks in the PL group with non-infected A549 cells, and the other two groups were injected with the same amount of 0.9% saline. The tumor volumes were monitored every week after tumor cell injection. After 4 weeks, the nude mice were sacrificed by Automated CO2 Delivery System. In brief, After the animals were put into the Automated CO_2_ Delivery System, the CO_2_ was infused into the box at the rate of 10–30% of the volume of the box per minute. When the animals were determined to be immobile, not breathing and pupil dilation (about 5 min), the CO_2_ was turned off and observed for 2 min to determine the death of the animal. The tumors were stripped and weighed, and immunohistochemistry (ki-67) and the TUNEL assay were performed. The study protocols were approved by the Animal Experimental Ethics Committee of the First Affiliated Hospital of Zhengzhou University.

### Immunohistochemistry

The processed paraffin sections were incubated with primary antibody against Ki-67 (1:50 dilution, AF1738; Beyotime, Beijing, China) followed by incubation with secondary antibodies according to the instructions.

### TUNEL assay

The TUNEL Assay Kit (C1088; Beyotime) was purchased, the experiment was conducted according to the manufacturer’s instructions, and the TUNEL staining was observed under a fluorescence microscope.

### Luciferase reporter assay

The wild-type (Wt) TGFBR1 3′-UTR and mutant type (Mt) TGFBR1 3′-UTR fragments were amplified by overlapping PCR. Then the two fragments were inserted into the pmirGLO promoter vector (Promega) to generate recombinant Wt TGFBR1 and Mt TGFBR1 plasmids, respectively. 293 T cells were seeded in 24-well plates and co-transfected with recombinant plasmids (Wt TGFBR1 or Mt TGFBR1) and miRNAs (miR-34b-3p mimics or miR-NC). Luciferase activities in each group were measured by a luminescence microplate reader (Berthold, Bad Wildbad, Germany).

### Western blotting

Western blotting was performed to detect protein expression after transfection. A549 and H1299 cells were lysed with RIPA lysis buffer (Beyotime), and total protein concentrations were detected by BCA Protein Assay Kit (Beyotime) followed by separation on 10% SDS-PAGE for western blot through incubation of the membrane with polyclonal rabbit anti-human TGFBR1 primary antibody (1: 2000 dilution) (Proteintech, WuHan, Hubei, China) at 4 °C overnight and subsequent incubation with HRP-conjugated secondary antibody (1: 5000 dilution) for 1 h at room temperature (Proteintech).

### Statistical analysis

SPSS 21.0 software was used for processing data. Each experiment was repeated three times. Data with normal distribution were presented as mean ± standard deviation. One-way analysis of variance was used to compare the difference among three or more groups; Student’s *t*-test was used to compare difference between two independent groups. *P* < 0.05 was considered to be statistically significant.

## Results

### PL upregulates miR-34b-3p expression in LC cell lines

**Fig. 1:**
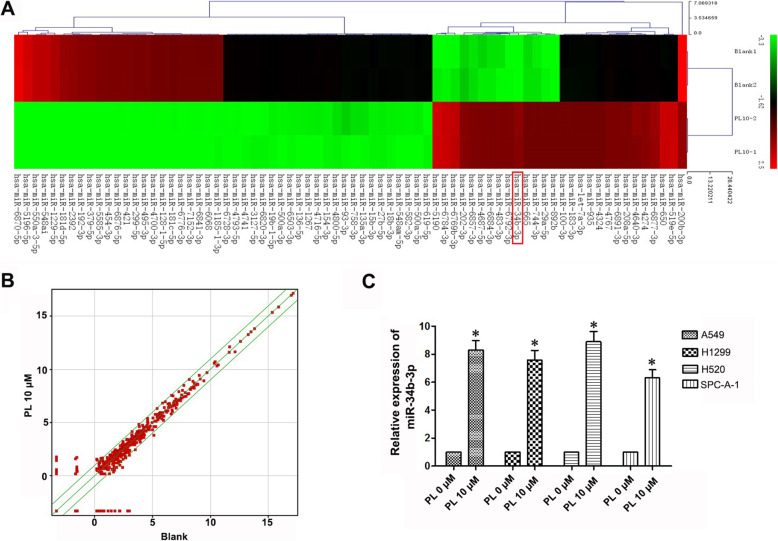
MiRNA chip and qPCR validation results. **a** Heat map of miRNA array in A549 cells with or without PL treatment. **b** Scatter plot of differentially expressed miRNAs with a more than 3-fold change. **c** Relative expression of miR-34b-3p with or without PL (0, 10 μM) treatment in different LC cells lines by qPCR. *, *P* < 0.05 compared with the control group

To study the anti-tumor mechanism of PL, miRNA microarray analysis was performed in A549 cells after treatment with PL. We found that there were 74 differentially expressed miRNAs with more than 3-fold changes, of which 27 were upregulated and 47 were downregulated. In addition, miR-34b-3p showed a 14-fold change increase in the 10 μM PL group compared with the 0 μM PL group (Table 1, Fig. 1a, b). To further confirm these results, miR-34b-3p expression was detected by quantitative PCR (qPCR) after total RNA extraction from PL-treated A549, H1299, H520, and SPC-A-1 NSCLC cells. Compared with cells in the 0 μM PL group, miR-34b-3p expression was consistently upregulated after 10 μM PL treatment in NSCLC cell lines (*P* < 0.05; Fig. 1c). These results showed that PL treatment increased the expression of miR-34b-3p.

**Table 1:** Summary of differentially expressed miRNAs by piperlongumine treatment using microarray

Name	miRNA location	Fold change	Regulation
hsa-miR-5190	chr18	33.42283	up
hsa-miR-6784-3p	chr17	31.10156	up
hsa-miR-6769b-3p	chr1	26.50146	up
hsa-miR-202-3p	chr10	14.70523	up
hsa-miR-34b-3p	chr11	14.6738	up
hsa-miR-6857-3p	chrX	13.53686	up
hsa-miR-6792-3p	chr19	13.4376	up
hsa-miR-483-3p	chr11	13.22746	up
hsa-miR-4687-5p	chr11	13.18184	up
hsa-miR-6884-3p	chr17	12.94226	up
hsa-miR-892b	chrX	11.49669	up
hsa-miR-744-3p	chr17	11.49669	up
hsa-miR-665	chr14	11.49669	up
hsa-miR-29a-5p	chr7	11.49669	up
hsa-miR-519e-5p	chr19	9.546338	up
hsa-miR-650	chr22	9.533337	up
hsa-miR-6877-3p	chr9	5.105282	up
hsa-miR-4274	chr4	4.374106	up
hsa-miR-4640-3p	chr6	3.953356	up
hsa-miR-6891-3p	chr6	3.834103	up
hsa-miR-208a-5p	chr14	3.791038	up
hsa-miR-4767	chrX	3.689143	up
hsa-miR-935	chr19	3.635668	up
hsa-miR-183-3p	chr7	3.635668	up
hsa-miR-100-3p	chr11	3.635668	up
hsa-let-7a-3p	chr9	3.635668	up
hsa-miR-4324	chr19	3.577973	up
hsa-miR-6870-5p	chr20	42.62034	down
hsa-miR-5196-5p	chr19	33.30788	down
hsa-miR-550a-3-5p	chr7	26.47759	down
hsa-miR-548ai	chr6	26.33067	down
hsa-miR-1229-5p	chr5	22.00426	down
hsa-miR-181d-5p	chr19	17.0129	down
hsa-miR-2392	chr14	15.64695	down
hsa-miR-192-3p	chr11	14.80487	down
hsa-miR-379-5p	chr14	14.55127	down
hsa-miR-5585-3p	chr1	13.2484	down
hsa-miR-454-3p	chr17	13.15125	down
hsa-miR-6876-5p	chr8	13.04917	down
hsa-miR-421	chrX	12.66647	down
hsa-miR-299-5p	chr14	12.45378	down
hsa-miR-495-3p	chr14	12.42371	down
hsa-miR-4700-3p	chr12	11.56176	down
hsa-miR-128-1-5p	chr2	11.53145	down
hsa-miR-6776-3p	chr17	11.18739	down
hsa-miR-7152-3p	chr10	11.18294	down
hsa-miR-1185-1-3p	chr14	11.16815	down
hsa-miR-6841-3p	chr8	11.15	down
hsa-miR-6068	chr1	11.07605	down
hsa-miR-181c-5p	chr19	11.07164	down
hsa-miR-6728-3p	chr1	3.467114	down
hsa-miR-4716-5p	chr15	3.448778	down
hsa-miR-154-3p	chr14	3.44251	down
hsa-miR-4800-5p	chr4	3.403111	down
hsa-miR-3127-5p	chr2	3.401971	down
hsa-miR-136-5p	chr14	3.384909	down
hsa-miR-4741	chr18	3.381469	down
hsa-miR-4793-5p	chr3	3.371059	down
hsa-miR-6503-3p	chr11	3.367946	down
hsa-miR-500a-3p	chrX	3.352311	down
hsa-miR-6820-3p	chr22	3.339673	down
hsa-miR-1267	chr13	3.338454	down
hsa-miR-19b-1-5p	chr13	3.284702	down
hsa-miR-500a-5p	chrX	3.183913	down
hsa-miR-200b-3p	chr1	3.150891	down
hsa-miR-93-3p	chr7	3.094811	down
hsa-miR-758-3p	chr14	3.094811	down
hsa-miR-619-5p	chr12	3.094811	down
hsa-miR-582-3p	chr5	3.094811	down
hsa-miR-548am-5p	chrX	3.094811	down
hsa-miR-27b-5p	chr9	3.094811	down
hsa-miR-18b-3p	chrX	3.094811	down
hsa-miR-15b-3p	chr3	3.094811	down
hsa-miR-135a-3p	chr3	3.094811	down

### PL treatment and upregulation of miR-34b-3p inhibits proliferation and invasion of A549 and H1299 LC cell lines

**Fig. 2:**
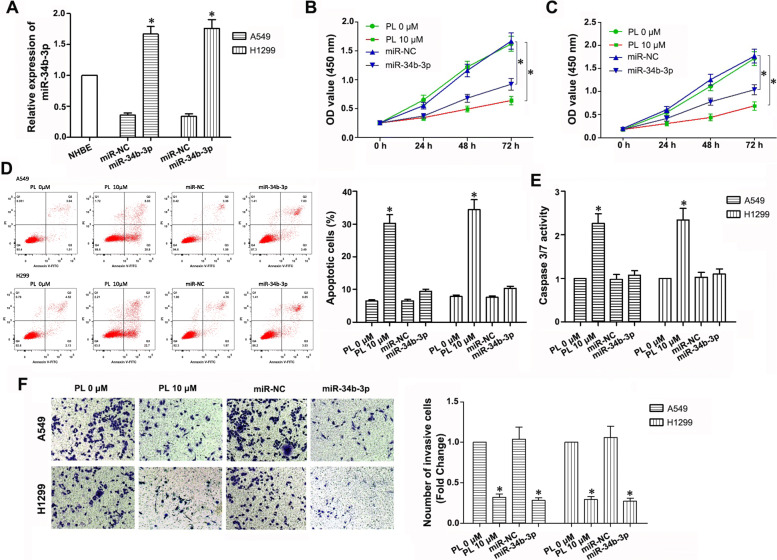
Effects of PL treatment and upregulation of miR-34b-3p on the proliferation, invasion, and apoptosis of NSCLC cells in vitro. **a** Relative expression of miR-34b-3p in normal human bronchial epithelial (NHBE) cells and lentivirus-infected A549 and H1299 cells using qPCR. **b**, **c** Optical density value was detected to represent the cell proliferation ability by CCK-8 Kit in the different groups. **d**, **e** Cell apoptosis was determined by flow cytometry and the caspase3/7 activity assay. **f** Cell invasion ability was detected 24 h after PL treatment by the transwell assay. A549 and H1299 were infected with miR-34b-3p lentivirus or treated with PL, then transwell assay was performed to detect invasive cell number. Invasive cells were stained with crystal violet and cell images were captured, then cell number were counted. All of the experiments were repeated three times

To evaluate the tumor-suppressing effects of miR-34b-3p and PL on LC, we constructed a lentiviral expression vector expressing miR-34b-3p or miR-negative control (NC), and then transfected separately into A549 and H1299 cells. Increased expression of miR-34b-3p after infection of the miR-34b-3p expressing lentivirus was found as demonstrated by qPCR, which indicated successful transfection (Fig. 2a). The CCK-8 assay showed that both upregulation of miR-34b-3p and PL inhibited cell proliferation (*P* < 0.05; Fig. 2b, c). In addition, a significant apoptosis-promoting effect was found after 10 μM PL treatment (*P* < 0.05); however, upregulation of miR-34b-3p had little effect on cell apoptosis (*P* > 0.05; Fig. 2d and e). In the transwell experiments, PL treatment and upregulation of miR-34b-3p effectively decreased the invasion ability of LC cells (*P* < 0.05; Fig. 2f). Overall, these data indicated that upregulating miR-34b-3p and PL treatment inhibited the proliferation and invasion of A549 and H1299 cells in vitro.

### miR-34b-3p overexpression and PL treatment inhibits the growth of A549 xenograft tumors in vivo

**Fig. 3:**
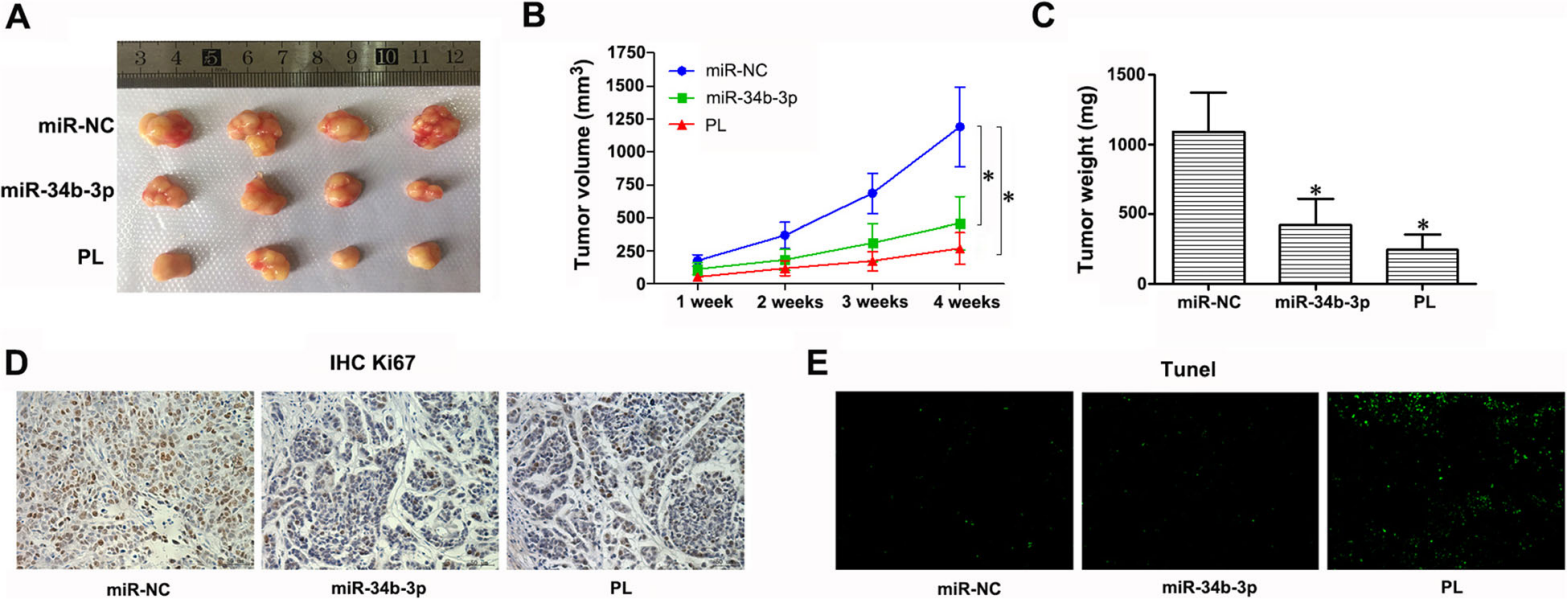
Effects of PL and miR-34b-3p on A549 xenograft mice. **a**, **b** The tumor volumes were measured once a week after transplantation, and the tumor nodules were stripped 4 weeks after transplantation after which the final volumes were measured. **c** The stripped tumor nodules were weighed. **d** Immunohistochemical staining of Ki-67. **e** Results of the TUNEL assay in three groups

To compare the effects of upregulated miR-34b-3p and PL on the proliferation of tumors in vivo, we performed a nude mouse xenograft experiment. The tumor volume was significantly reduced in the inoculated nude mice of miR-34b-3p group and PL group compared with mice of the miR-NC group after 2, 3, and 4 weeks (*P* < 0.05; Fig. 3a, b). In addition, the tumor weight in the miR-34b-3p and PL groups showed a significant decrease (*P* < 0.05; Fig. 3c). Immunohistochemical staining of Ki-67 in the xenograft tumor tissue showed that the positive staining of Ki-67 in the tumor cells was significantly lower in the miR-34b-3p and PL groups than that in the miR-NC group (*P* < 0.05; Fig. 3d). We also investigated the effect of miR-34b-3p and PL on tumor apoptosis using the terminal deoxynucleotidyl transferase dUTP nick end labeling (TUNEL) assay. As shown in Fig. 3e, the number of apoptotic cells in the PL group was significantly higher than that in the miR-NC and miR-34b-3p group. Taken together, the in vivo results were consistent with in vitro assays indicating that both PL and miR-34b-3p inhibits the proliferation of NSCLC cells and xenograft tumor growth, but only PL promotes tumor cell apoptosis.

### miR-34b-3p targets TGFBR1

**Fig. 4:**
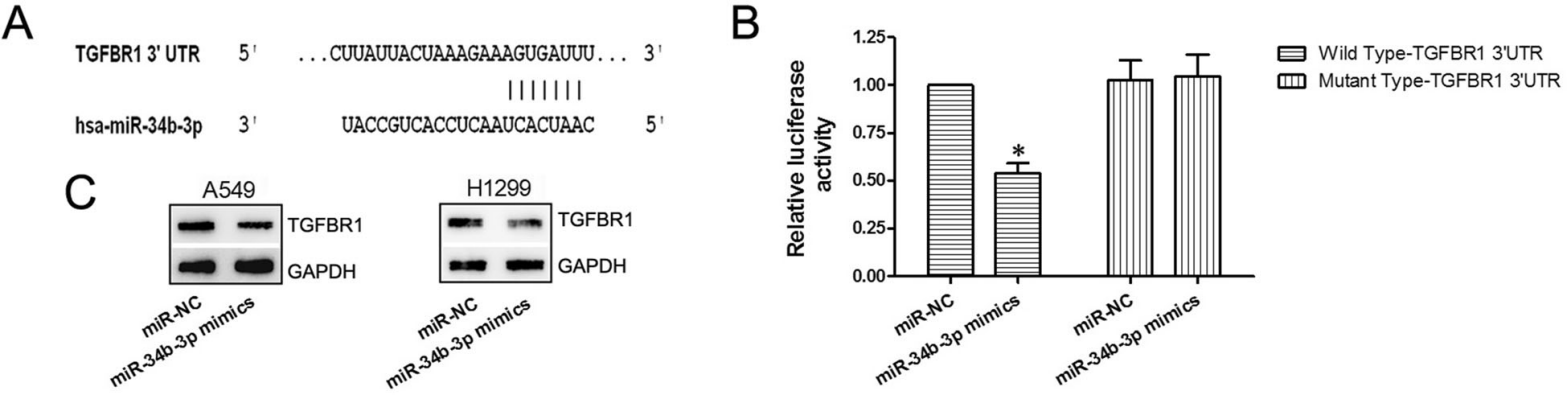
TGFBR1 is a target of miR-34b-3p. **a** TargetScan bioinformatics analysis results. **b** Relative luciferase activity results of the dual-luciferase reporter assay. **c** Relative protein expression level of TGFBR1 after a 48 h transfection of miR-NC or miR-34b-3p mimics by western blotting. GAPDH served as the internal reference

The result of bioinformatics analysis through TargetScan indicated that the TGFBR1 mRNA shared a complementary region with miR-34b-3p, and thus may be a potential target of miR-34b-3p (Fig. 4a). We verified this through dual-luciferase reporter assays and found that in the miR-34b-3p mimics and wild-type pmirGLO co-transfection group, the fluorescence activity was significantly reduced compared to the other three groups (*P* < 0.05; Fig. 4b), suggesting that miR-34b-3p bound to the TGFBR1 mRNA 3’UTR and decreased firefly luciferase activity. Moreover, Western blotting showed an apparent decrease in TGFBR1 protein expression after miR-34b-3p mimics transfection (*P* < 0.05; Fig. 4c). Taken together, these data showed that TGFBR1 is a target gene of miR-34b-3p.

### Overexpression of TGFBR1 attenuates the inhibitory effects of PL on the proliferation and migration of A549 and H1299 cells

**Fig. 5:**
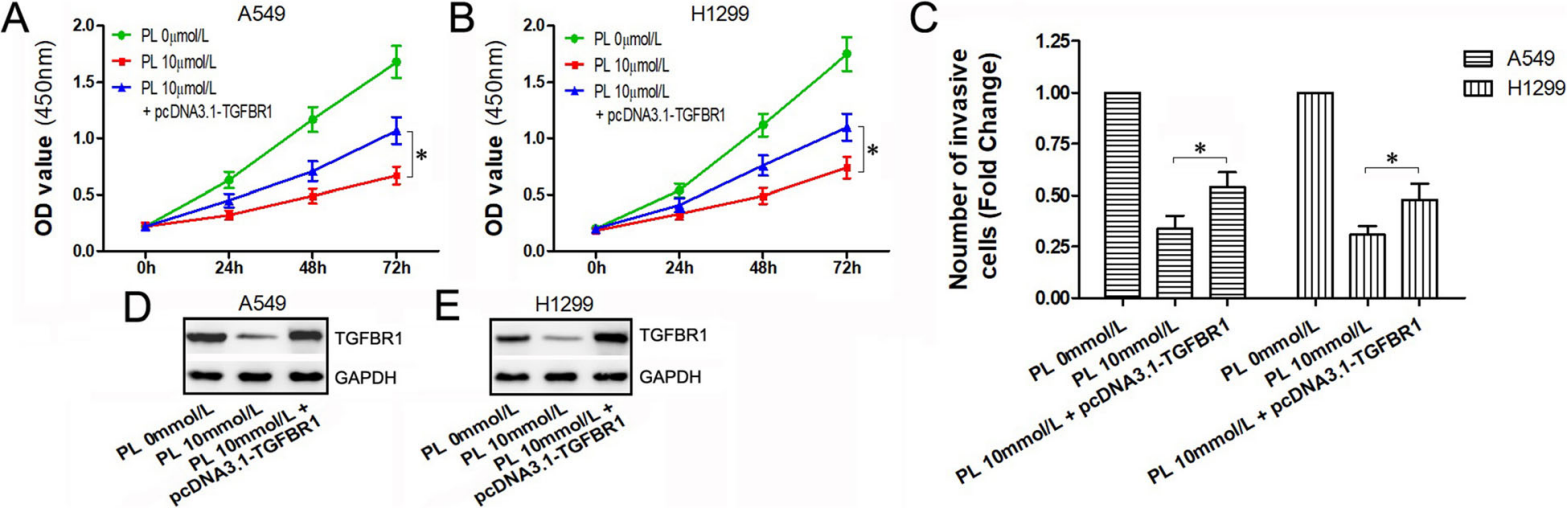
Overexpression of TGFBR1 lacking 3′ UTR may restore the effects of PL. **a**, **b** Cell growth curve and optical density value were obtained from the CCK-8 assay in cells treated with PL alone, PL combined with TGFBR1 lacking 3′ UTR, and blank control. **c** Cell migration ability was evaluated by the transwell assay in cells treated with PL alone, PL combined with TGFBR1 lacking 3′ UTR, and blank control. **d**, **e** Relative protein expression level of TGFBR1 was detected by western blotting after 48 h of PL treatment or TGFBR1 transfection

To further demonstrate the role of PL/miR-34b-3p/TGFBR1 axis in LC cells, we constructed an expression vector of TGFBR1 lacking the 3 ‘UTR (named pcDNA3.1-TGFBR1) which was transfected into PL-treated A549 and H1299 cells (combination group). The CCK-8 assay showed that compared to cells with PL treatment alone, the combination group showed few inhibitory effects on both A549 and H1299 cells (Fig. 5a, b), indicating that overexpression of TGFBR1 partially restored the inhibitory effects on PL-induced cell proliferation. In addition, the migration effects of PL on tumor cells were attenuated after TGFBR1 overexpression (Fig. 5c). Western blot analysis showed that PL treatment alone significantly decreased TGFBR1 protein expression which was rarely affected in the combination group (Fig. 5d, e).

## Discussion

There is evidence that PL has anti-tumor effects in the progression of LC through various pathways, including the Akt [25] and nuclear factor-kappa B (NF-κB) signaling pathways [26]. PL suppresses NF-κB signaling pathway by directly binding to the DNA binding site of NF-κB p50 subunit and decreasing nuclear translocation of NF-κB p65 subunit, and inhibits Akt signaling pathway by suppressing phosphorylation of Akt in lung tumor cells. Moreover, PL can regulate expressions of c-Myc-regulated miRNAs (miR-27a, miR-20a, and miR-17) by decreasing c-Myc via an epigenetic pathway [14]. Here, we also supposed that PL may upregulate miR-34b-3p by some transcription factors induced transcriptional regulation. However, the related mechanisms are quite complicated and involve several pathways, thus needing further exploration. If the anti-tumor effects of PL are confirmed in the clinic, it will certainly be beneficial to patients. In this study, we showed that PL inhibited the proliferation of NSCLC cell lines (A549 and H1299), upregulated the expression of miR-34b-3p, and reduced the expression of TGFBR1. It has been reported lower miR-34b-3p level in cancers, such as LC, pancreatic cancer, and prostate cancer [14, 27, 28]. miR-34b-3p plays a role in tumor inhibition, such as pancreatic cancer [29], oral cancer [30], bladder cancer [31], cholangiocarcinoma [32], breast cancer [22], cervical cancer [33], ovarian cancer [23], and colorectal cancer [34]. In LC studies, the abnormal expression of miR-34b-3p in H1299 and A549 cells was shown to represses cell proliferation, cell cycle progression, and promote cell apoptosis by targeting cyclin-dependent kinase 4 [35]. Consistently, our study found that PL/miR-34b-3p/TGFBR1 axis influenced cell growth, invasion, and apoptosis of A549 and H1299 cells. TGFBR1 is a component of the transforming growth factor beta (TGF-β)/SMAD signaling pathway, which can regulate cell growth, differentiation, and migration [36]. TGF-β (tumor promoter or inhibitor) plays a key role in tumorigenesis [37]. TGFBR1 participates in cellular processes, differentiation, and apoptosis [38, 39]. The activation or overexpression of TGFBR1 is found in various types of cancer. For example, it can enhance the migration and invasion of breast cancer cells and promote the invasion and metastasis of colorectal cancer [39–42]. Furthermore, TGFBR1 affects tumor invasion and metastasis through the epithelial-mesenchymal pathway [43]. Together, these results indicate that TGFBR1 inhibition may be a novel therapeutic target for tumor treatment, consistent with previous results showing that downregulation of TGFBR1 suppresses cell proliferation, migration, and invasion in NSCLC [44]. In this study, the expression of TGFBR1 was affected after PL treatment and TGFBR1 knockdown inhibited cell proliferation and promoted cell apoptosis, consistent with PL-induced upregulation of miR-34b-3p expression. The overexpression of TGFBR1 repressed the proliferation and invasion of A549 and H1299 cells, suggesting that PL affected growth and apoptosis of NSCLC cells, and upregulated miR-34b-3p by targeting TGFBR1.

## Conclusions

PL upregulates the expression of miR-34b-3p in NSCLC cell lines and inhibit cell proliferation and migration through TGFBR1 signaling pathway.

## Supplementary Information

**Additional file 1.** Fig. 4C-A549-GAPDH: The original Fig. 4C GAPDH image of WB in A549 cells. Fig. 4C-A549-GAPDH-L: The original Fig. 4C GAPDH image with text labels in A549 cells. Fig. 4C-A549-TGFBR1: The original Fig. 4C TGFBR1 image of WB in A549 cells. Fig. 4C-A549-TGFBR1-L: The original Fig. 4C TGFBR1 image with text labels in A549 cells. Fig. 4C-H1299-GAPDH: The original Fig. 4C GAPDH image of WB in H1299 cells. C- Fig. 4H1299-GAPDH-L: The original Fig. 4C GAPDH image with text labels in H1299 cells. C- Fig. 4H1299-TGFR1: The original Fig. 4C TGFR1 image of WB in H1299 cells. C- Fig. 4H1299-TGFR1-L: The original Fig. 4C TGFR1 image with text labels in H1299 cells. Fig. 5 DE-GAPDH: The original GAPDH image in Figure 5D and 5E. Fig. 5 DE-GAPDH-L: The original GAPDH image with text labels in Figure 5D and 5E. Fig. 5 DE-TGFBR1: The original TGFBR1 image in Figure 5D and 5E. Figure 5 DE-TGFBR1-L: The original TGFBR1 image with text labels in Figure 5D and 5E.

**Additional file 2.**

**Additional file 3.**

**Additional file 4.**

**Additional file 5.**

**Additional file 6.**

**Additional file 7.**

**Additional file 8.**

## Data Availability

Not applicable
