# Supplementary material for: Piperlongumine inhibits the growth of non-small cell lung cancer cells via the miR-34b-3p/TGFBR1 pathway
Source: BMC Complement Med Ther. 2021 Jan 7;21:15. doi: 10.1186/s12906-020-03123-y (PMC7791704; doi:10.1186/s12906-020-03123-y)

**Figure legends:**

Fig. 4C-A549-GAPDH: The original Fig. 4C GAPDH image of WB in A549 cells.


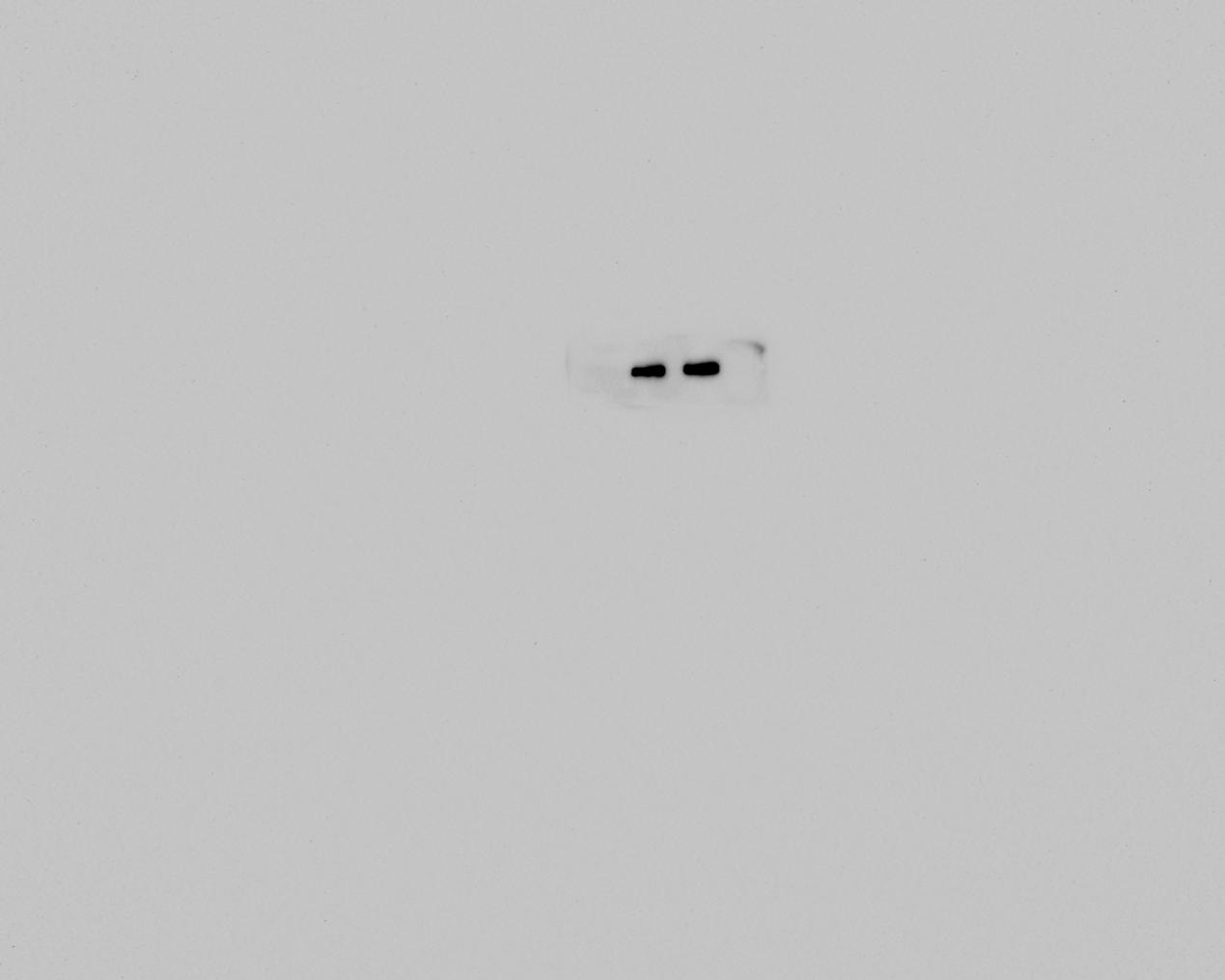


Fig. 4C-A549-GAPDH-L: The original Fig. 4C GAPDH image with text labels in A549 cells.


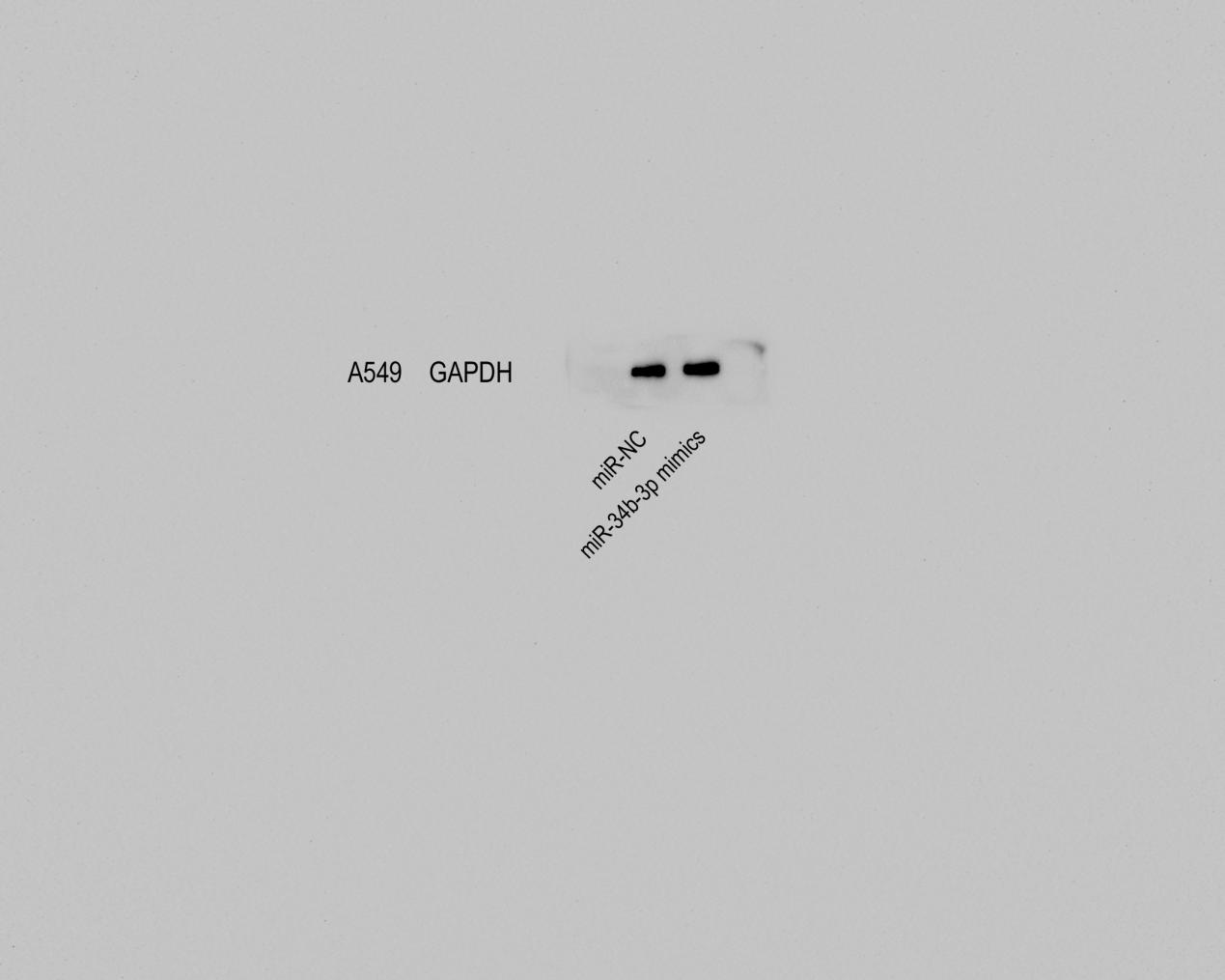


Fig. 4C-A549-TGFBR1: The original Fig. 4C TGFBR1 image of WB in A549 cells.


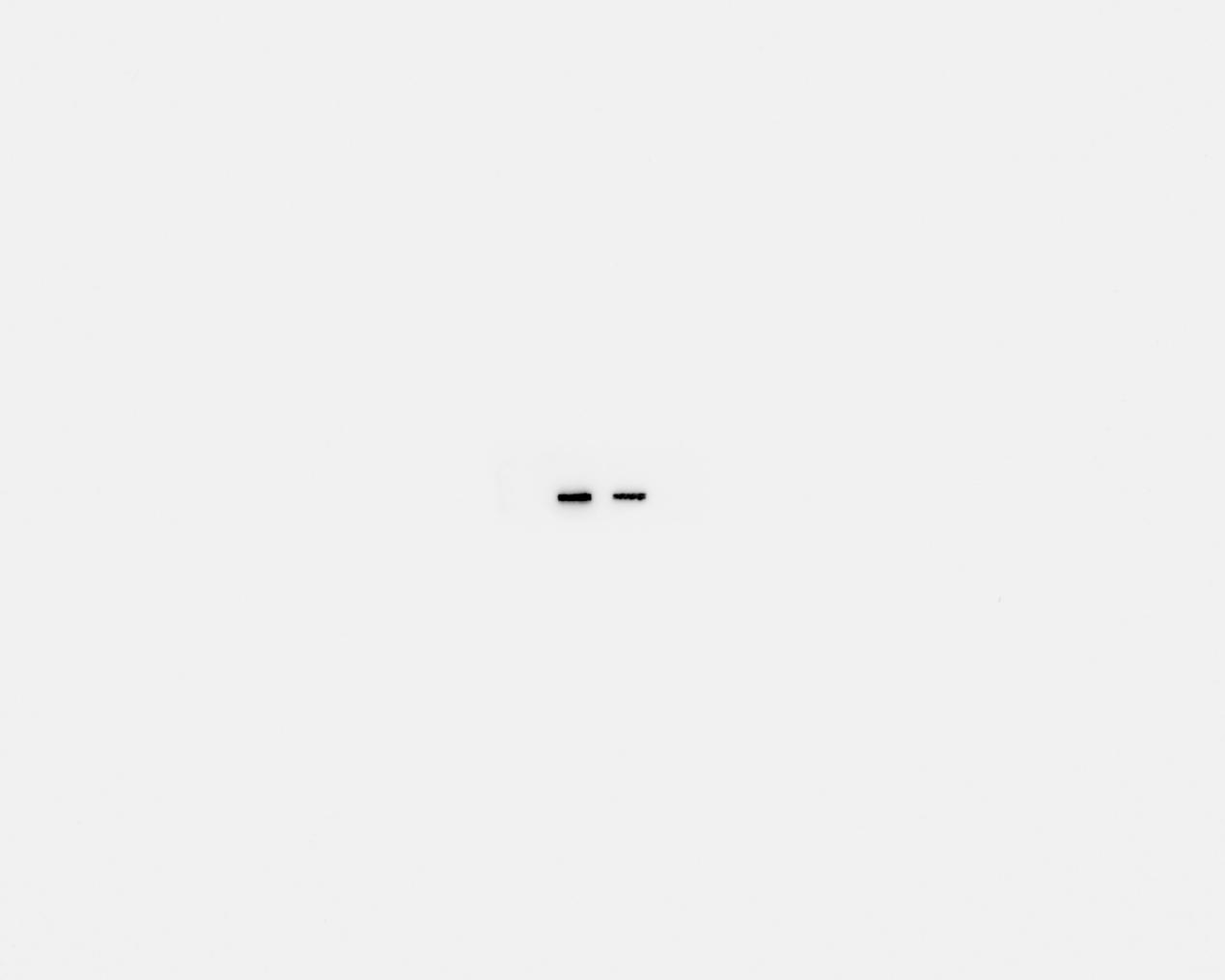


Fig. 4C-A549-TGFBR1-L: The original Fig. 4C TGFBR1 image with text labels in A549 cells


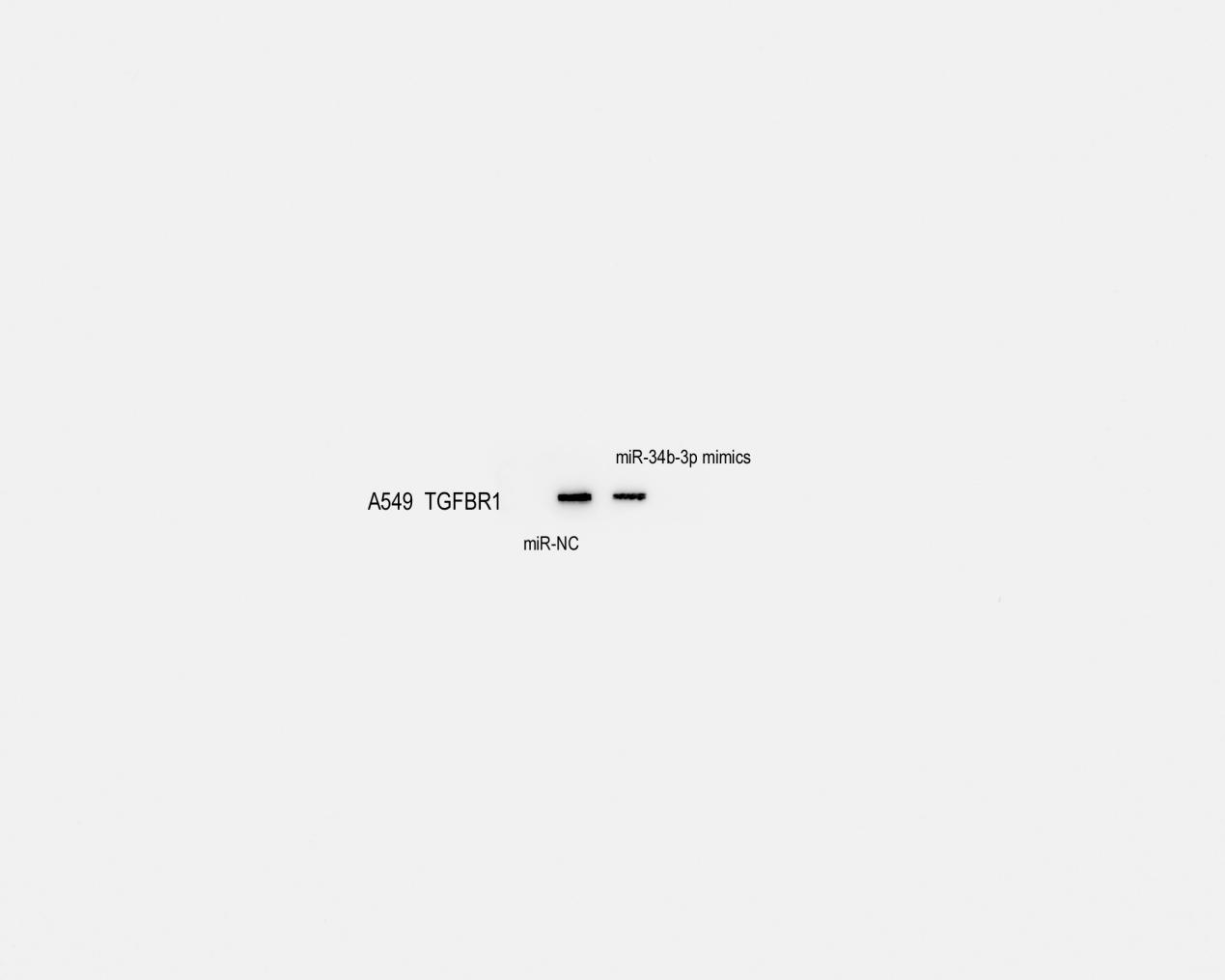


Fig. 4C-H1299-GAPDH: The original Fig. 4C GAPDH image of WB in H1299 cells.


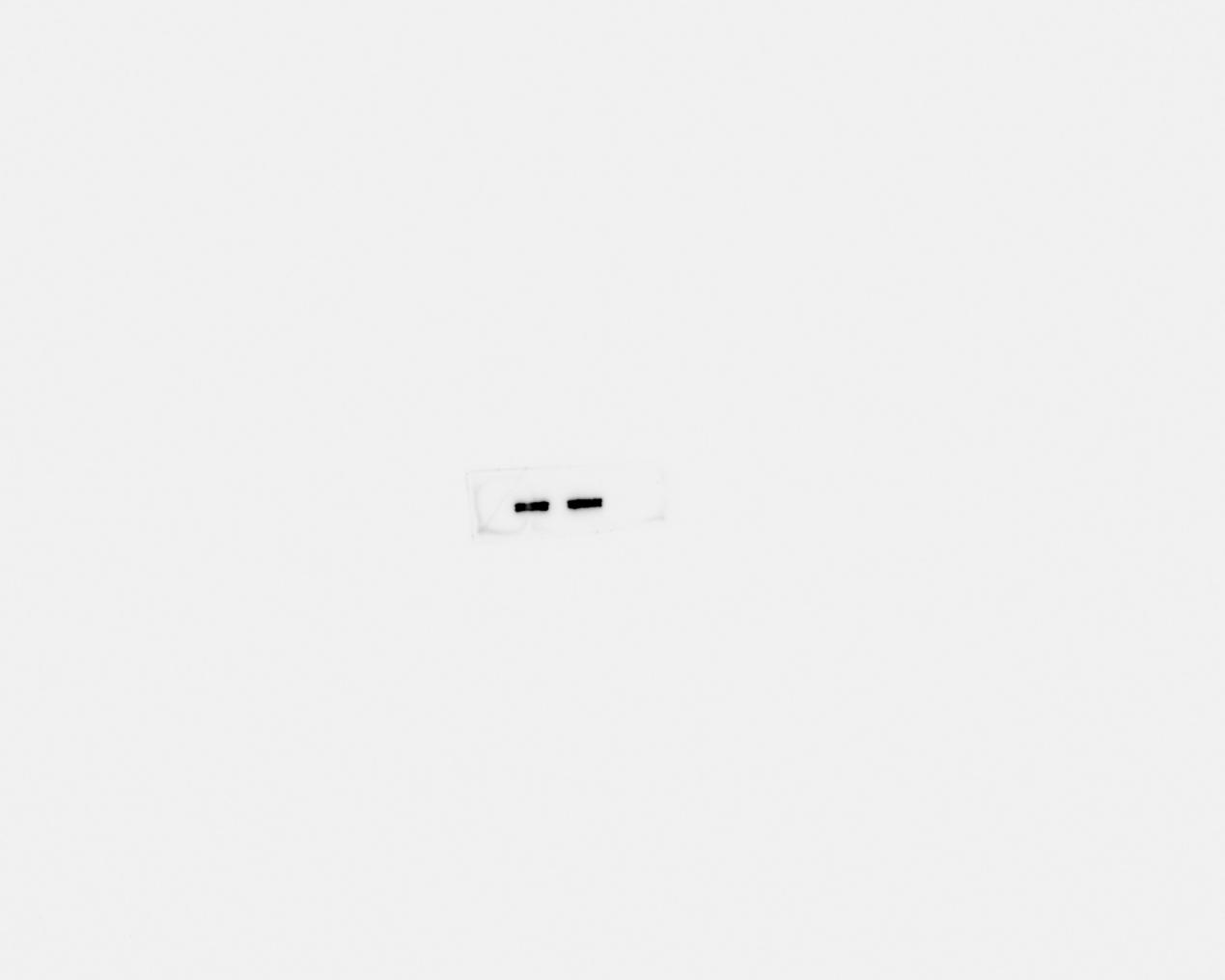


Fig. 4C- H1299-GAPDH-L: The original Fig. 4C GAPDH image with text labels in H1299 cells.
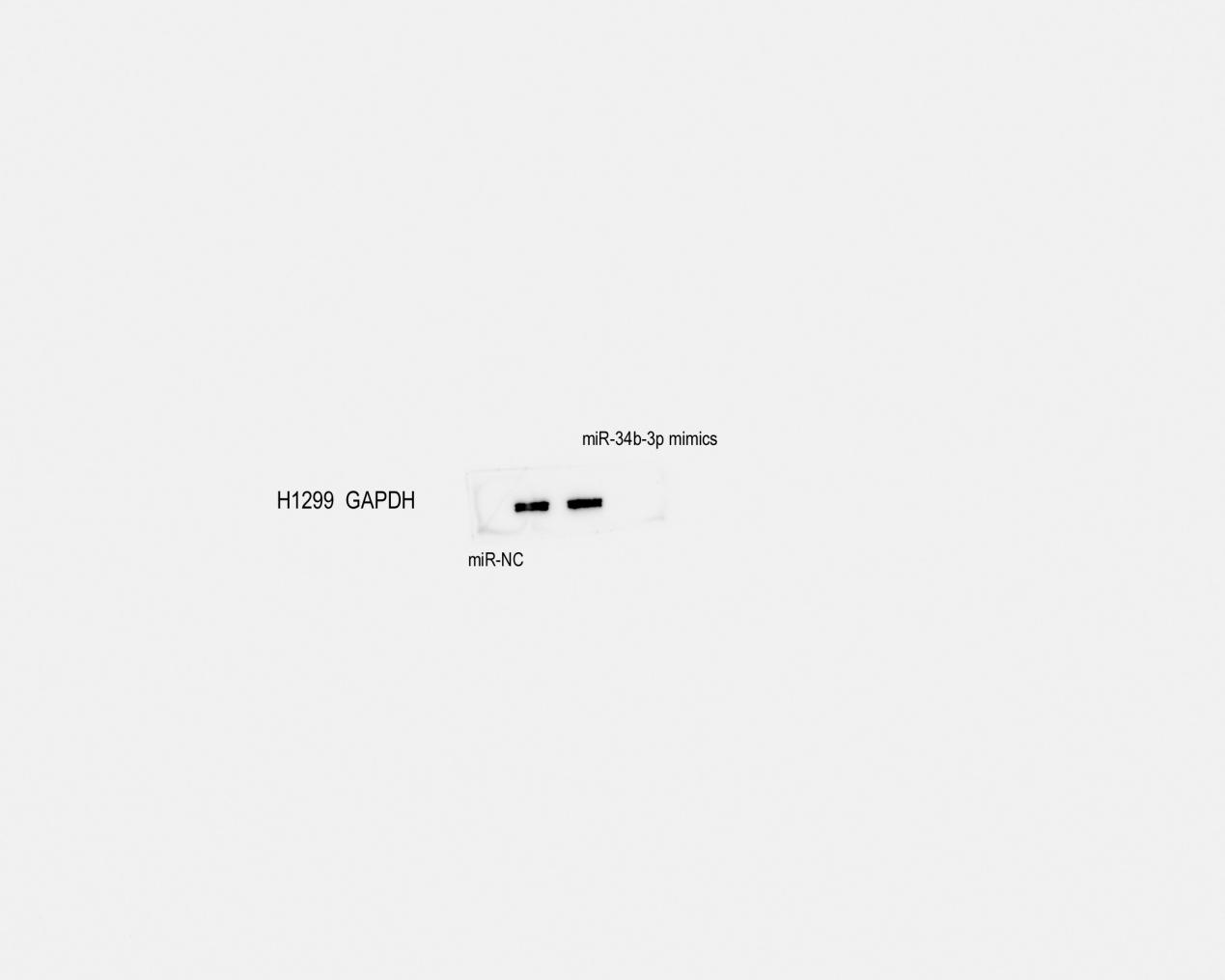


Fig. 4C- H1299-TGFR1: The original Fig. 4C TGFR1 image of WB in H1299 cells.


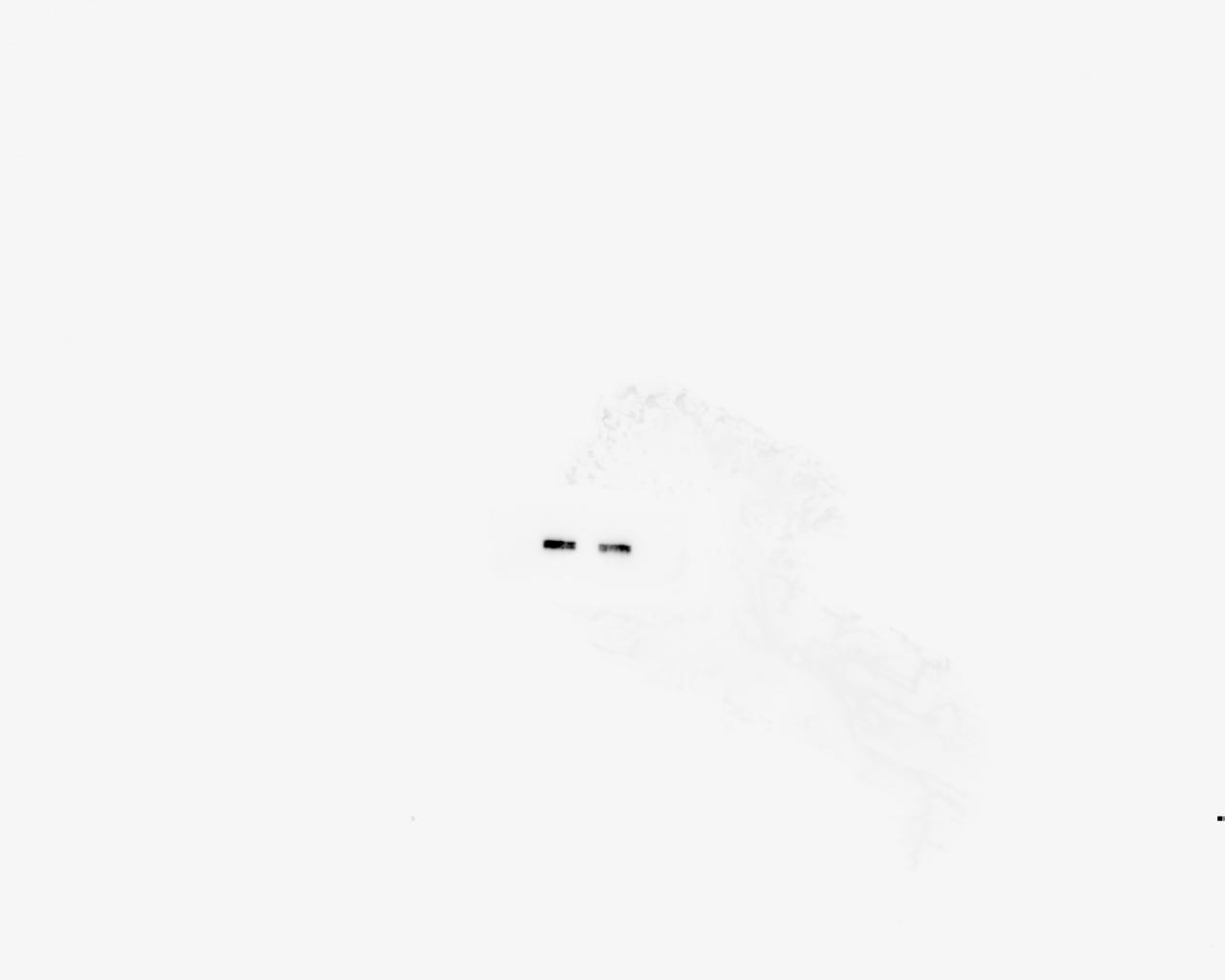


Fig. 4C- H1299-TGFR1-L: The original Fig. 4C TGFR1 image with text labels in H1299 cells


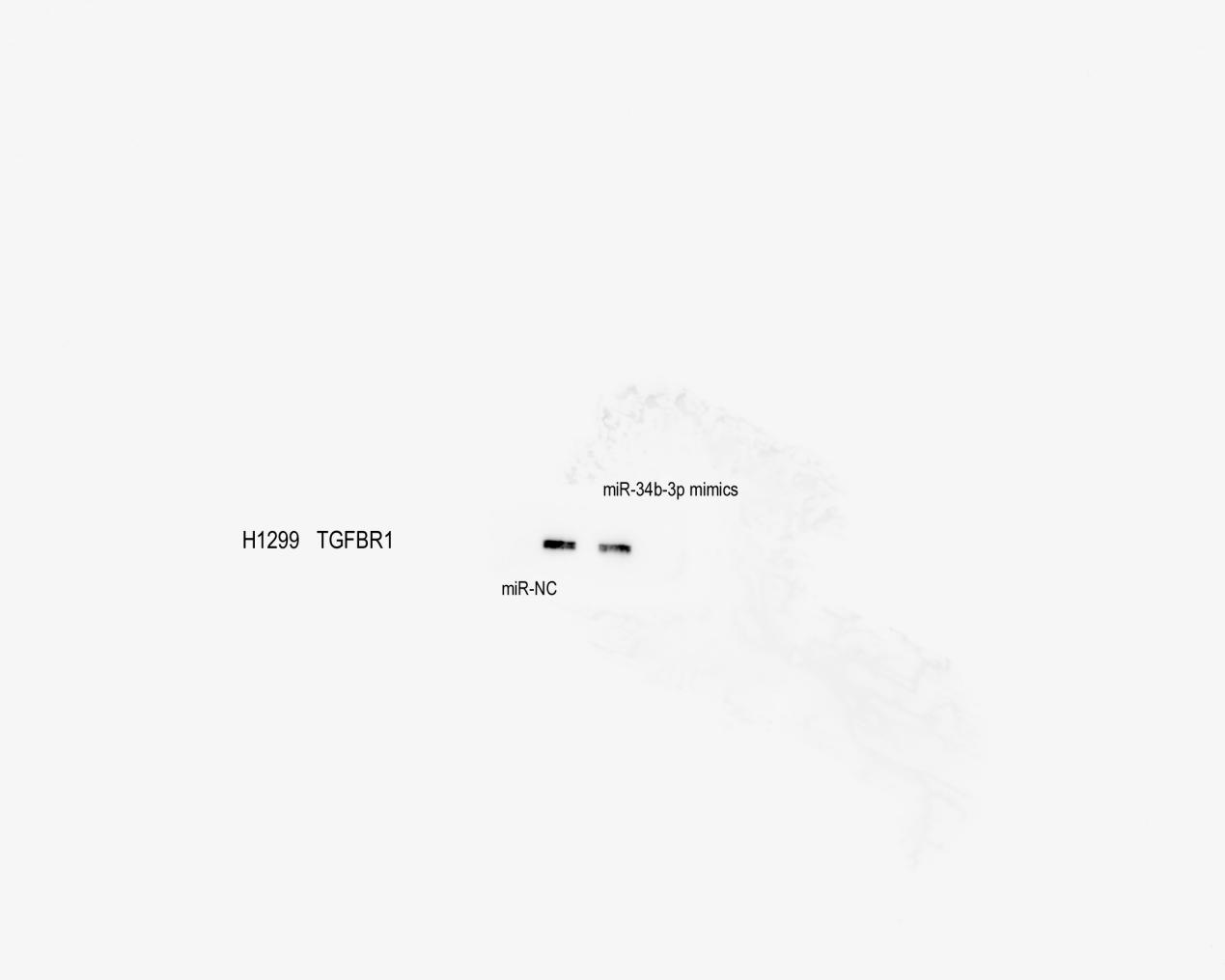


Fig. 5DE-GAPDH: The original GAPDH image in Figure 5D and 5E.


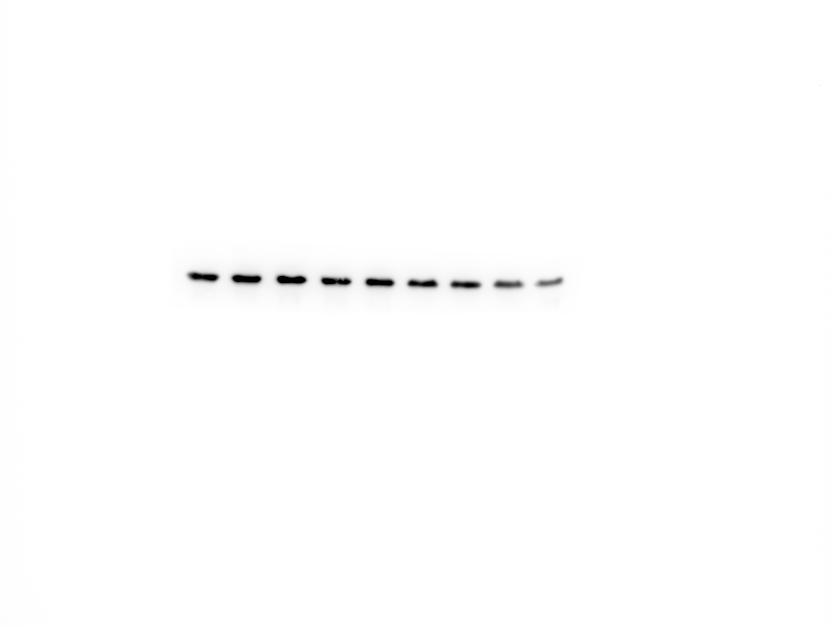


Fig. 5DE-GAPDH-L: The original GAPDH image with text labels in Figure 5D and 5E.
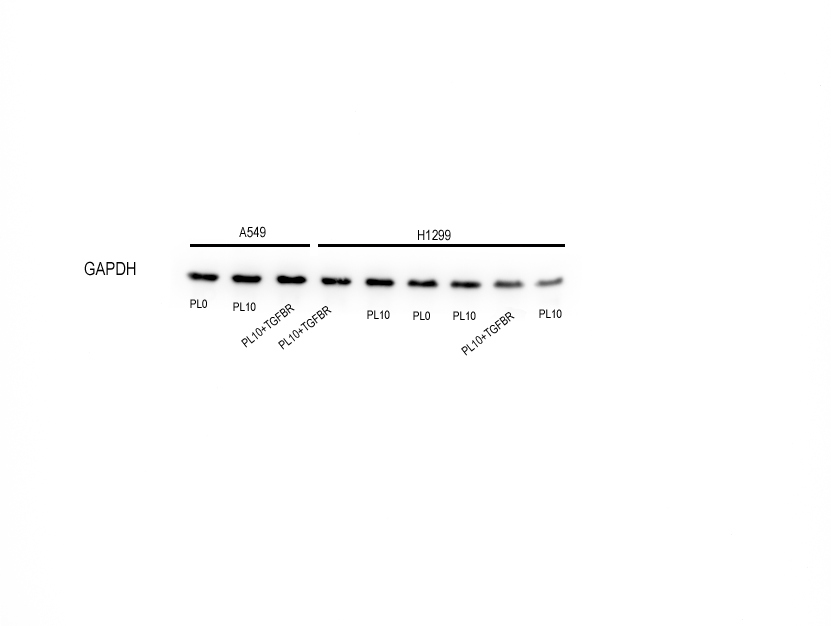


Fig. 5DE-TGFBR1: The original TGFBR1 image in Figure 5D and 5E.


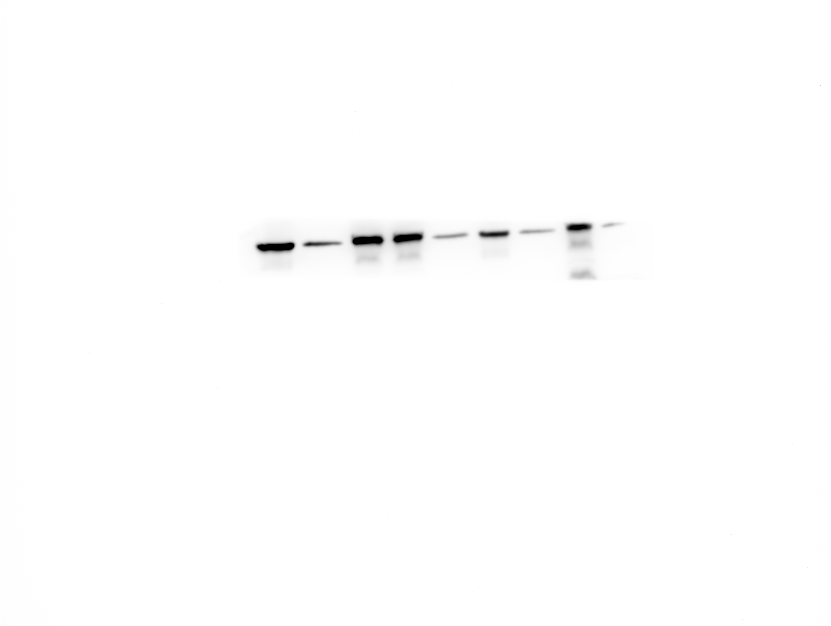


Fig. 5DE-TGFBR1-L: The original TGFBR1 image with text labels in Figure 5D and 5E.


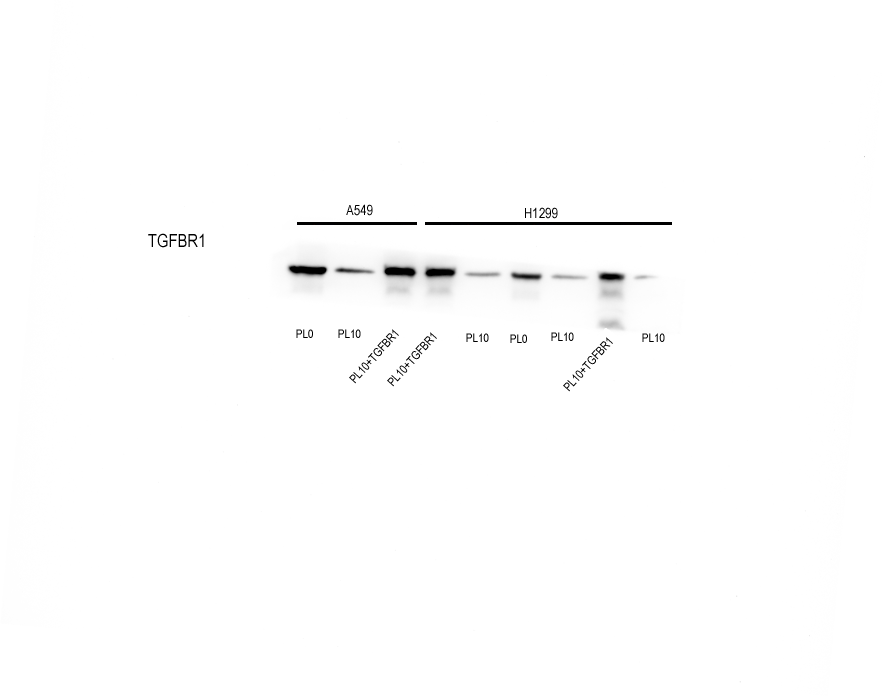

Supplement: Supplementary file 1 — Additional file 1. Fig. 4C-A549-GAPDH: The original Fig. 4C GAPDH image of WB in A549 cells. Fig. 4C-A549-GAPDH-L: The original Fig. 4C GAPDH image with text labels in A549 cells. Fig. 4C-A549-TGFBR1: The original Fig. 4C TGFBR1 image of WB in A549 cells. Fig. 4C-A549-TGFBR1-L: The original Fig. 4C TGFBR1 image with text labels in A549 cells. Fig. 4C-H1299-GAPDH: The original Fig. 4C GAPDH image of WB in H1299 cells. C- Fig. 4H1299-GAPDH-L: The original Fig. 4C GAPDH image with text labels in H1299 cells. C- Fig. 4H1299-TGFR1: The original Fig. 4C TGFR1 image of WB in H1299 cells. C- Fig. 4H1299-TGFR1-L: The original Fig. 4C TGFR1 image with text labels in H1299 cells. Fig. 5 DE-GAPDH: The original GAPDH image in Figure 5D and 5E. Fig. 5 DE-GAPDH-L: The original GAPDH image with text labels in Figure 5D and 5E. Fig. 5 DE-TGFBR1: The original TGFBR1 image in Figure 5D and 5E. Figure 5 DE-TGFBR1-L: The original TGFBR1 image with text labels in Figure 5D and 5E. [file 12906_2020_3123_MOESM1_ESM.zip › Figure legendsR3.docx]
